# Efficacy of anti-epidermal growth factor antibody rechallenge in *RAS/BRAF* wild-type metastatic colorectal cancer: a multi-institutional observational study

**DOI:** 10.1007/s00432-024-05893-1

**Published:** 2024-07-27

**Authors:** Koshiro Fukuda, Hiroki Osumi, Yuri Yoshinami, Akira Ooki, Atsuo Takashima, Takeru Wakatsuki, Hidekazu Hirano, Izuma Nakayama, Kota Ouchi, Ryoichi Sawada, Shota Fukuoka, Mariko Ogura, Daisuke Takahari, Keisho Chin, Hirokazu Shoji, Natsuko Okita, Ken Kato, Naoki Ishizuka, Narikazu Boku, Kensei Yamaguchi, Eiji Shinozaki

**Affiliations:** 1 Department of Gastroenterological Chemotherapy, Cancer Institute Hospital, Japanese Foundation for Cancer Research, Tokyo, Japan; 2 Department of Gastroenterology, Graduate School of Medicine, The University of Tokyo, Tokyo, Japan; 3 Gastrointestinal Medical Oncology Division, National Cancer Center Hospital, Tokyo, Japan; 4 Department of Medical Oncology, Tohoku University Hospital, Miyagi, Japan; 5 Division of Gastroenterology and Hepatology, Department of Internal Medicine, The Jikei University School of Medicine, Tokyo, Japan; 6 Center for Digital Transformation of Healthcare, Graduate School of Medicine, Kyoto University, Kyoto, Japan; 7 Department of Oncology and General Medicine, IMSUT Hospital, The Institute of Medical Science, The University of Tokyo, Tokyo, Japan

**Keywords:** Anti-EGFR monoclonal antibody, Circulating tumor DNA, *RAS/BRAF* wild-type, Rechallenge, Response to first-line anti-EGFR monoclonal antibody

## Abstract

**Purpose:**

To investigate circulating tumor DNA (ctDNA) *RAS* mutant (MT) incidence before salvage-line treatment and the clinicopathological features and molecular biological factors associated with the efficacy of anti-epithelial growth factor receptor (EGFR) monoclonal antibody (mAb) rechallenge for tissue *RAS*/*BRAF* wild type (WT) metastatic colorectal cancer (mCRC).

**Methods:**

This multi-institutional retrospective observational study included 74 patients with mCRC with tissue *RAS/BRAF* WT refractory to first-line chemotherapy containing anti-EGFR mAb. ctDNA *RAS* status was assessed using the OncoBEAM™ *RAS* CRC Kit. We explored the clinicopathological features associated with ctDNA *RAS* status and the factors related to anti-EGFR mAb rechallenge efficacy in multivariate Cox proportional hazard regression.

**Results:**

The incidence of *RAS* MT in ctDNA was 40.5% (30/74), which was associated with primary tumor resection (*P* = 0.016), liver metastasis (*P* < 0.001), and high tumor marker levels (*P* < 0.001). Among the 39 patients treated with anti-EGFR mAb rechallenge, those with ctDNA *RAS* WT showed significantly longer progression-free survival (PFS) than those with ctDNA *RAS* MT (median 4.1 vs. 2.7 months, hazard ratio [HR] = 0.39, *P* = 0.045). Patients who responded to first-line anti-EGFR mAb showed significantly longer PFS (HR = 0.21, *P* = 0.0026) and overall survival (OS) (HR = 0.23, *P* = 0.026) than those with stable disease.

**Conclusions:**

The incidence of ctDNA *RAS* MT mCRC was 40.5%, which was associated with liver metastases and high tumor volumes. Anti-EGFR mAb rechallenge may be effective for patients with mCRC who responded to first-line chemotherapy containing anti-EGFR mAb. No patients with *RAS* MT in ctDNA responded to anti-EGFR mAb rechallenge.

**Supplementary Information:**

The online version contains supplementary material available at 10.1007/s00432-024-05893-1.

## Introduction

The anti-epithelial growth factor receptor (EGFR) monoclonal antibodies (mAbs) cetuximab and panitumumab improve survival in patients with *RAS* wild-type (WT) metastatic colorectal cancer (mCRC), but do not yield a significant survival benefit in patients with right-sided or *RAS* mutant (MT) mCRC (Di Nicolantonio et al. 2021). Therefore, several guidelines recommend the use of anti-EGFR mAb as a first-line treatment for patients with left-sided *RAS/BRAF* WT mCRC (Hashiguchi et al. 2020). However, a recent report suggested that anti-EGFR mAbs might be a treatment option for patients with mCRC without baseline ctDNA alteration, such as *KRAS*, *NRAS*, *PTEN*, and extracellular domain *EGFR* mutations, *HER2*, *MET* amplifications, *ALK*, *RET*, and *NTRK1* fusions, even in right-sided tumors (Shitara et al. 2024). Moreover, according to the ESMO Clinical Practice Guideline for mCRC, right-sided tumors might benefit less in terms of progression-free survival (PFS) and overall survival (OS) from treatment with anti-EGFR mAb compared with left-sided tumors, but anti-EGFR mAb may be effective in terms of tumor shrinkage regardless of tumor sidedness (Cervantes et al. 2023). While *RAS* status is typically assessed prior to the initiation of systemic chemotherapy, most patients develop acquired resistance even after an initial response to chemotherapy containing anti-EGFR mAb (Misale et al. 2012; Siravegna et al. 2016), indicating changes in cancer clones and/or genetic status. Therefore, changes in the *RAS* status following anti-EGFR mAb administration remain to be characterized. The development of liquid biopsy technology, especially for the assessment of circulating tumor DNA (ctDNA), has enabled the monitoring of real-time tumor-derived genetic alterations. Mutant *RAS* clones have been reported to emerge in the blood during anti-EGFR mAb treatment and to decline treatment discontinuation, suggesting that the clonal evolution relates to clinical progression (Morelli et al. 2015; Siravegna et al. 2015; Siena et al. 2018; Parseghian et al. 2019). Anti-EGFR mAb rechallenge is defined as the re-administration of anti-EGFR mAb after an anti-EGFR mAb-free period in patients showing resistance to prior chemotherapy.

The concept of anti-EGFR mAb rechallenge was first reported by Santini et al. They assessed the efficacy of cetuximab rechallenge in patients with mCRC and reported promising clinical outcomes (Santini et al. 2012). However, they did not appropriately distinguish between anti-EGFR mAb reintroduction and rechallenge. The CRICKET trial was the first multicenter phase II study to evaluate the efficacy of cetuximab rechallenge. The ad-hoc analysis of this study revealed that patients with mCRC with ctDNA *RAS* WT prior to the anti-EGFR mAb rechallenge had longer PFS than those with ctDNA *RAS* MT (Cremolini et al. 2019). An ad-hoc analysis of several clinical trials on anti-EGFR mAb rechallenge also showed an association between survival and *RAS* status in ctDNA (JACCRO CC-08 and 09, E-rechallenge) (Osawa et al. 2018; Sunakawa et al. 2020). In terms of combination therapy as anti-EGFR mAb rechallenge and other treatments besides irinotecan, the CAVE trial investigated rechallenge with cetuximab plus avelumab in refractory *RAS* WT mCRC; this combined therapy was well tolerated, and patients with *RAS*/*BRAF* WT ctDNA had significantly longer median PFS and OS than did those with *RAS*/*BRAF* MT ctDNA (Martinelli et al. 2021, Ciardiello D et al. 2022). The CHRONOS study, an open-label, single-arm, phase II trial, was the first trial to prospectively evaluate the efficacy of anti-EGFR mAb rechallenge based on the mutational status of ctDNA and showed that their efficacy was resumed (Sartore-Bianchi et al. 2022). These results suggest that evaluation of the *RAS* mutational status of ctDNA may help to select candidates for anti-EGFR mAb rechallenge.

Several previous studies have reported the incidence of ctDNA *RAS* MT before salvage-line treatment and a post-hoc pooled analysis of the CAVE and VELO trial investigated that 75.2% of patients retained *RAS*/*BRAF* WT ctDNA before rechallenge with anti-EGFR mAb (Ciardiello et al. 2023, Germani et al. 2024), as well as the clinicopathological features and predictors of the efficacy of anti-EGFR mAb rechallenge, except for pretreatment ctDNA *RAS* status (Ciardiello et al. 2024). However, it remains unclear whether these clinicopathological features would be significant factors in daily clinical practice. It is also necessary to identify other promising predictors of response and survival for anti-EGFR mAb rechallenge.

Therefore, this study aimed to investigate the incidence of ctDNA *RAS* MT before the initiation of salvage-line treatment and explore the clinicopathological features and molecular biological factors associated with the efficacy of anti-EGFR mAb rechallenge for tissue *RAS*/*BRAF* WT mCRC.

## Methods

### Study population

From June 2021 to December 2022, this multi-institutional retrospective observational study consecutively enrolled patients with mCRC from four institutions who had tissue *RAS/BRAF* WT at first-line chemotherapy with anti-EGFR mAb, for whom ctDNA *RAS* status was examined after second- or later-line chemotherapy without anti-EGFR mAb. Anti-EGFR mAb rechallenge was defined as readministration of anti-EGFR mAb after an anti-EGFR mAb-free period in patients showing resistance to prior anti-EGFR mAb. Some of these patients received anti-EGFR mAb rechallenge after ctDNA examination.

### Methods of ctDNA examination

We used the OncoBEAM™ *RAS* CRC Kit, which detects 34 mutations in *KRAS*/*NRAS* codons 12, 13, 59, 61, 117, and 146 at an allele frequency of 0.01% (Nikanjam et al. 2022).

### Clinical data and assessment

We collected the following clinical data from the electronic medical records: age, sex, primary tumor site (right-sided colon [cecum, ascending colon, or transverse colon] or left-sided colon [descending colon, sigmoid colon, or rectum]), metastatic site, primary tumor resection, treatment lines and regimens immediately before ctDNA examination, serum tumor markers (carcinoembryonic antigen [CEA] and carbohydrate antigen 19 − 9 [CA19-9]), tissue *RAS*/*BRAF* status at diagnosis, ctDNA *RAS* status. For patients who received a rechallenge of anti-EGFR mAb, information on anti-EGFR mAb-free interval (aEFI), responses, PFS, and OS were also collected. The response was evaluated according to the RECIST guidelines (v1.1) (Schwartz et al. 2016). The response rate (RR) was calculated as the proportion of patients showing complete response (CR) or partial response (PR) among those with measurable disease, and the disease control rate (DCR) was that of patients showing CR, PR, or stable disease (SD). PFS was defined as the time from initiation of the anti-EGFR mAb rechallenge to either the first objective disease progression or death from any cause, and OS was defined as the time from initiation of the anti-EGFR mAb rechallenge to death from any cause.

### Statistical analyses

We investigated the proportion of patients with ctDNA *RAS* MT and the ctDNA *RAS* mutation sites and compared the clinicopathological characteristics between patients with ctDNA *RAS* MT and those with ctDNA *RAS* WT. Categorical variables were compared using Fisher’s exact test, and continuous variables were compared using two-sample *t*-tests. We also evaluated the clinical outcomes of patients who received anti-EGFR mAb rechallenge.

Time-to-event was assessed using the Kaplan–Meier method and compared using log-rank tests. The clinicopathological factors associated with PFS and OS, including ctDNA *RAS* status, were explored using univariate and multivariate analyses. Possible confounders, including sex, age, primary tumor location, metastatic sites (liver, lung, peritoneum, lymph nodes), aEFI (< 12 months or ≥ 12 months), treatment lines at the time of sampling (fourth or later, or third), ctDNA *RAS* status, and anti-EGFR mAb best response (PR or SD) in first-line chemotherapy containing ant-EGFR mAb were selected for univariate analysis and the factors with a significant level below 0.1 were analyzed by multivariate Cox proportional hazard regression analysis (backward stepwise methods). The association between aEFI and PFS was investigated using the Spearman’s rank correlation coefficient. All statistical tests were two-sided, and significance was set at *P* < 0.05. Statistical analyses were performed using EZR statistical software (Saitama Medical Center, Jichi Medical University, Saitama, Japan), which is a modified version of the R commander designed to add specific statistical functions commonly used in biostatistics (Kanda 2013).

## Results

### Cohort characteristics

In total, 74 patients with mCRC with tissue *RAS/BRAF* WT who were refractory to prior chemotherapies, including fluoropyrimidines, oxaliplatin, irinotecan, and anti-EGFR mAb, were enrolled. Regarding all patient characteristics, the median age was 63 years (range: 36–83 years), and the cohort included 41 (55.4%) males and 33 (44.6%) females. A total of 65 patients (87.8%) had a primary lesion in the left-sided colon, while 9 (12.2%) had lesions in the right-sided colon. In terms of metastatic sites, 57 patients (77.0%) had liver metastases, 51 (68.9%) had lung metastases, 40 (54.1%) had lymph node metastases, and 25 (33.8%) had peritoneal metastases. In addition, 37 patients (50.0%) had metastases in more than 3 organs, and 47 (63.5%) underwent resection of primary lesions. Regarding tumor markers, the median CEA and CA19-9 levels were 119.1 ng/mL and 88.8 U/mL, respectively.

**Fig. 1:**
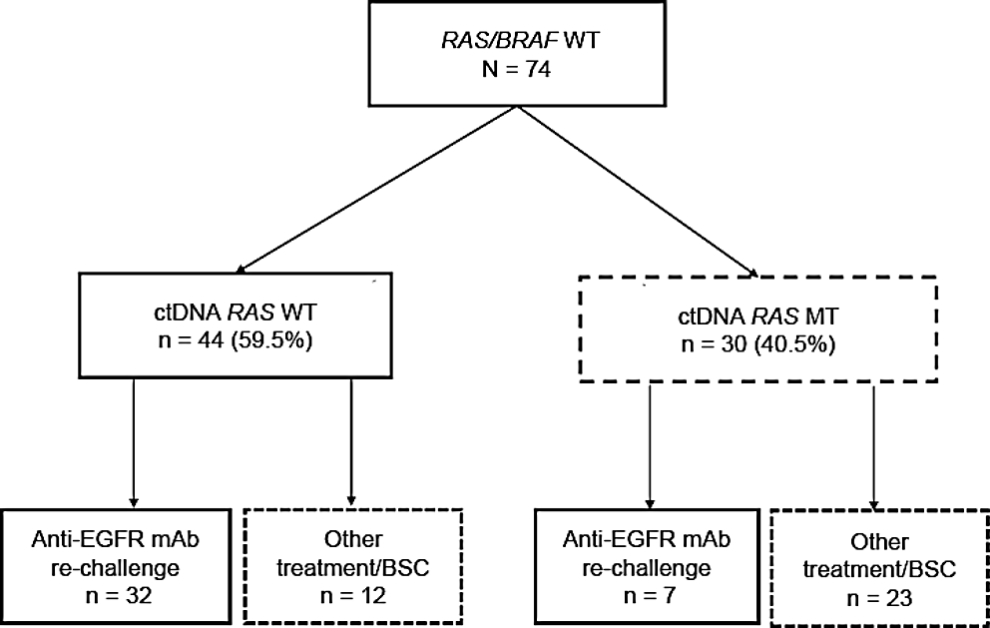
STROBE flow diagram. WT, wild type; ctDNA, circulating tumor DNA; MT, mutant type; EGFR, epidermal growth factor receptor; mAb, monoclonal antibody; BSC, best supportive care

Regarding ctDNA examination results, 30 patients (40.5%) were MT, and 44 (59.5%) were WT (Fig. 1). The most common site of *RAS* mutation detected in ctDNA was *KRAS* codon 61 (*n* = 17, 33%), followed by *KRAS* codon 12 (*n* = 14, 27%), and *NRAS* codon 61 (*n* = 12, 24%). Clinicopathological features, according to the ctDNA *RAS* status examined after prior chemotherapy, are listed in Table 1.

**Table 1:** Patient characteristics (*N* = 74)

Characteristics, *n* (%)	All patients (*N* = 74)	ctDNA *RAS* WT (*n* = 44, 59.5%)	ctDNA *RAS* MT (*n* = 30, 40.5%)	*P* value
**Age at enrollment, years**				
Median [range]	63 [36–83]	65 [48–83]	62 [36–82]	0.072
**Sex**				
Male	41 (55.4)	27 (61.4)	14 (46.7)	0.24
Female	33 (44.6)	17 (38.6)	16 (53.3)	
**Primary site**				
Right-sided colon	9 (12.2)	4 (9.1)	5 (16.7)	0.68
Left-sided colon	65 (87.8)	40 (90.9)	25 (83.3)	
**Metastatic site**				
Liver				
Present	57 (77.0)	27 (61.4)	30 (100)	< 0.001
Absent	17 (23.0)	17 (38.6)	0 (0)	
Lung				
Present	51 (68.9)	30 (68.2)	21 (70)	1
Absent	23 (31.1)	14 (31.8)	9 (30)	
Lymph node				
Present	40 (54.1)	22 (50)	18 (60)	0.48
Absent	34 (45.9)	22 (50)	12 (40)	
Peritoneum				
Present	25 (33.8)	15 (34.1)	10 (33.3)	1
Absent	49 (66.2)	29 (65.9)	20 (66.7)	
**Number of metastatic sites**				
1–2 organs	37 (50)	25 (56.8)	12 (40)	0.24
3 organs and more	37 (50)	19 (43.2)	18 (60)	
**Resection of primary tumor**				
Yes	47 (63.5)	33 (75)	14 (46.7)	0.016
No	27 (36.5)	11 (25)	16 (53.3)	
**Re-challenge treatment lines**				
Third line	25 (33.8)	16 (36.4)	9 (30)	0.62
Fourth line or later	49 (66.2)	28 (63.6)	21 (70)	
**Tumor markers**				
CEA, median [range] (ng/mL)	119.1 [2.2–12670.2]	64.3 [2.2–3023.6]	305.6 [2.5–12670.2]	< 0.001
CA19-9, median [range] (U/mL)	88.8 [1.0–7820.0]	32.5 [1.0–6927.8]	504.5 [1.0–7820.0]	< 0.001

CEA: Carcinoembryonic antigen

CA19-9: Carbohydrate antigen 19 − 9

ctDNA: Circulating tumor DNA

WT: Wild type

MT: Mutant type

*RAS*: Rat sarcoma viral oncogene homolog

Significant differences in the frequency of ctDNA *RAS* MT were observed between patients who underwent resection of the primary tumor and those who did not (29.8% [14/47] vs. 59.3% [16/27], *P* = 0.016) and between patients without and with liver metastasis (0% [0/17] vs. 52.6% [30/57], *P* < 0.001). Patients with ctDNA *RAS* MT showed higher CEA levels than those with ctDNA *RAS* WT (median CEA: 1123 vs. 191 ng/mL, *P* = 0.01). No significant differences were observed in ORR and DCR during the first-line chemotherapy containing anti-EGFR mAb between patients with ctDNA *RAS* WT and MT.

### Clinical outcomes of anti-EGFR mAb rechallenge

Of the 74 patients with mCRC, 39 (52.7%) received anti-EGFR mAb rechallenge as monotherapy or in combination with irinotecan (Supplemental Table 1). Their median age was 63 years (36–82); 25 (64.1%) were male, and 36 (92.3%) had primary lesions in the left-sided colon. In terms of metastatic sites, 28 patients (71.8%) had liver metastases, 27 (69.2%) had pulmonary metastases, 17 (43.6%) had lymph node metastases, 14 (35.9%) had peritoneal metastases, and 27 (69.2%) underwent primary lesion resection. Considering treatment lines for anti-EGFR mAb rechallenge, 14 patients (33.8%) received this rechallenge as a third-line treatment. In terms of ctDNA status, 32 patients (82.1%) had ctDNA *RAS* WT and 7 (17.9%) had ctDNA *RAS* MT. The median anti-EGFR mAb-free interval was 14.9 months (5.1–82.6 months). Among the 39 patients, the response rate during first-line chemotherapy containing anti-EGFR mAb in the 33 patients who had target lesions was 81.8% (27/33), whereas aEFI was assessed in 37. Two patients received first-line chemotherapy combined with anti-EGFR mAb in other hospitals, and we could not find out the final day of the first-line anti-EGFR mAb.

The ORR and DCR of these 39 patients who were treated with anti-EGFR mAb rechallenge were 12.1% (4/33) and 48.5% (16/33), respectively. Their median PFS was 3.7 months (95% confidence interval [CI]: 2.5–5.1), and their median OS was 10.4 months (95% CI, 6.6–not available).

**Fig. 2:**
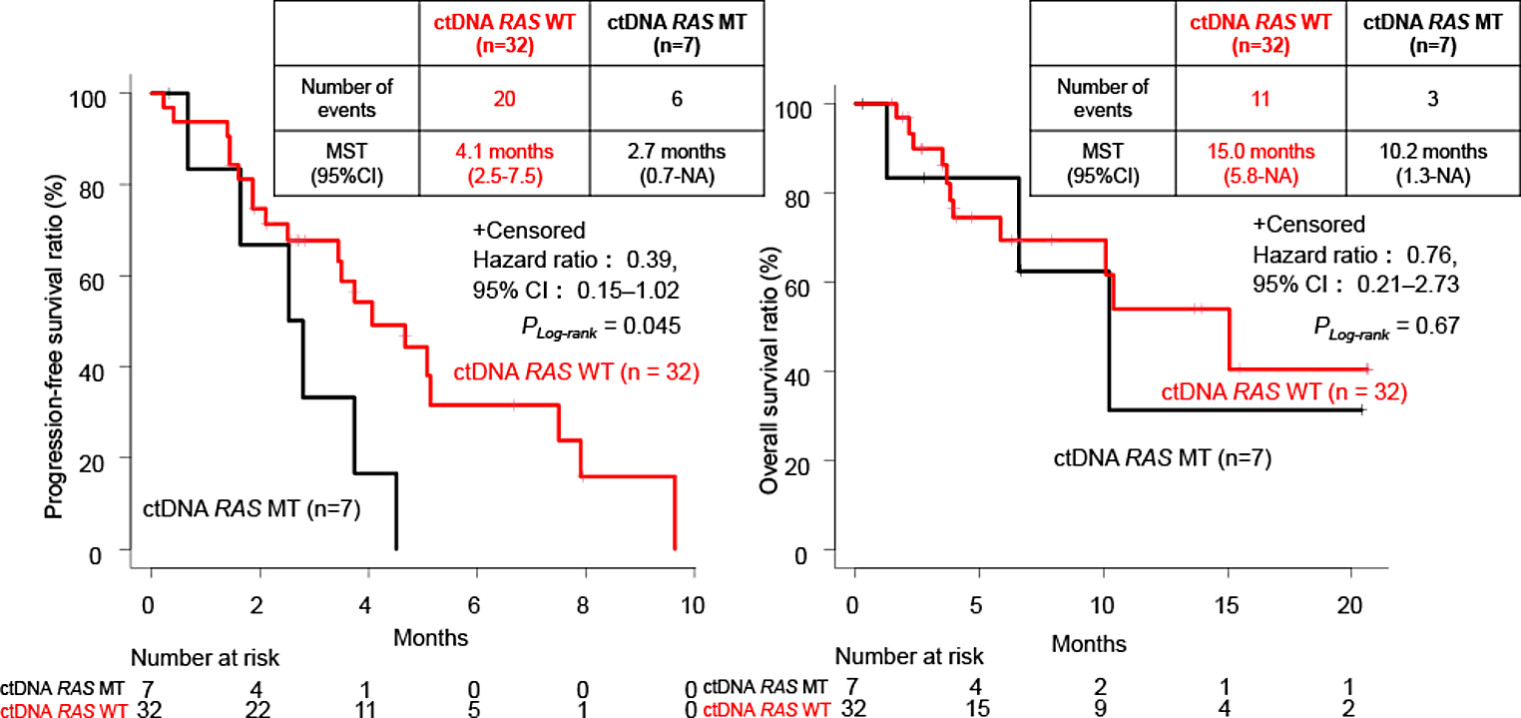
Progression-free survival (PFS) and overall survival (OS) after anti-epidermal growth factor receptor (EGFR) monoclonal antibody (mAb) rechallenge for patients with metastatic colorectal cancer (mCRC) with circulating tumor DNA (ctDNA) *RAS* wild-type (WT) or mutant (MT) (*n* = 39). MST, median survival time; CI, confidence interval; NA, not applicable

Of the 32 patients with mCRC with ctDNA *RAS* WT, 5 (15.6%) achieved PR, whereas none of the 7 patients with ctDNA *RAS* MT achieved PR. Patients with ctDNA *RAS* WT had significantly longer PFS than those with ctDNA *RAS* MT (median PFS: 4.1 vs. 2.7 months; hazard ratio [HR], 0.39; 95% CI, 0.15–1.02, *P*_*Log−rank*_ = 0.045). There was no significant difference in OS between two groups (HR, 0.82; 95% CI, 0.23–2.97, *P*_*Log−rank*_ = 0.76) (Fig. 2, Supplemental Table 3).

**Fig. 3:**
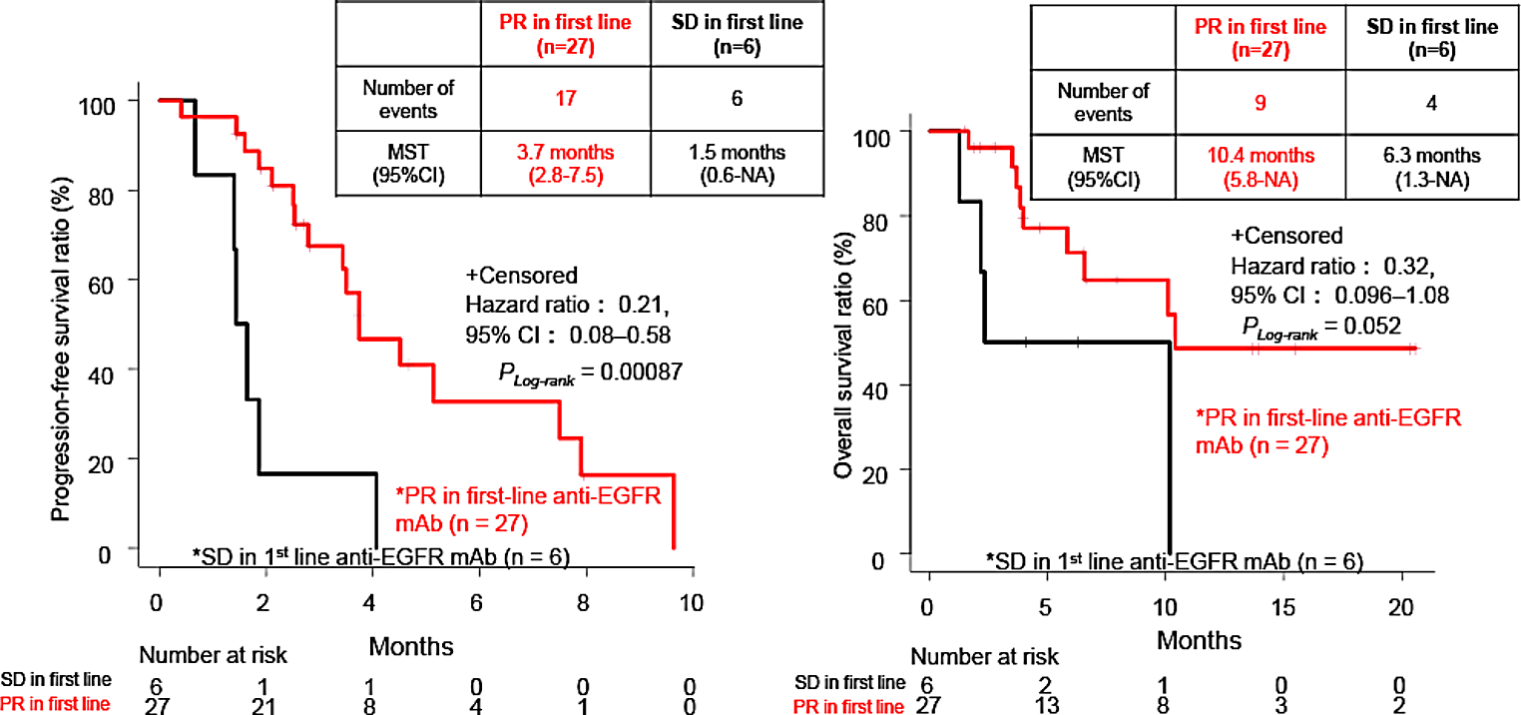
Progression-free survival (PFS) and overall survival (OS) after anti-epidermal growth factor receptor (EGFR) monoclonal antibody (mAb) for metastatic colorectal cancer (mCRC) patients showing partial response (PR) or stable disease (SD) in first-line anti-EGFR mAb (*n* = 33). *PR/SD in first line: patients with mCRC who had PR or SD during first-line anti-EGFR mAb. MST, median survival time; CI, confidence interval; NA, not applicable

**Fig. 4:**
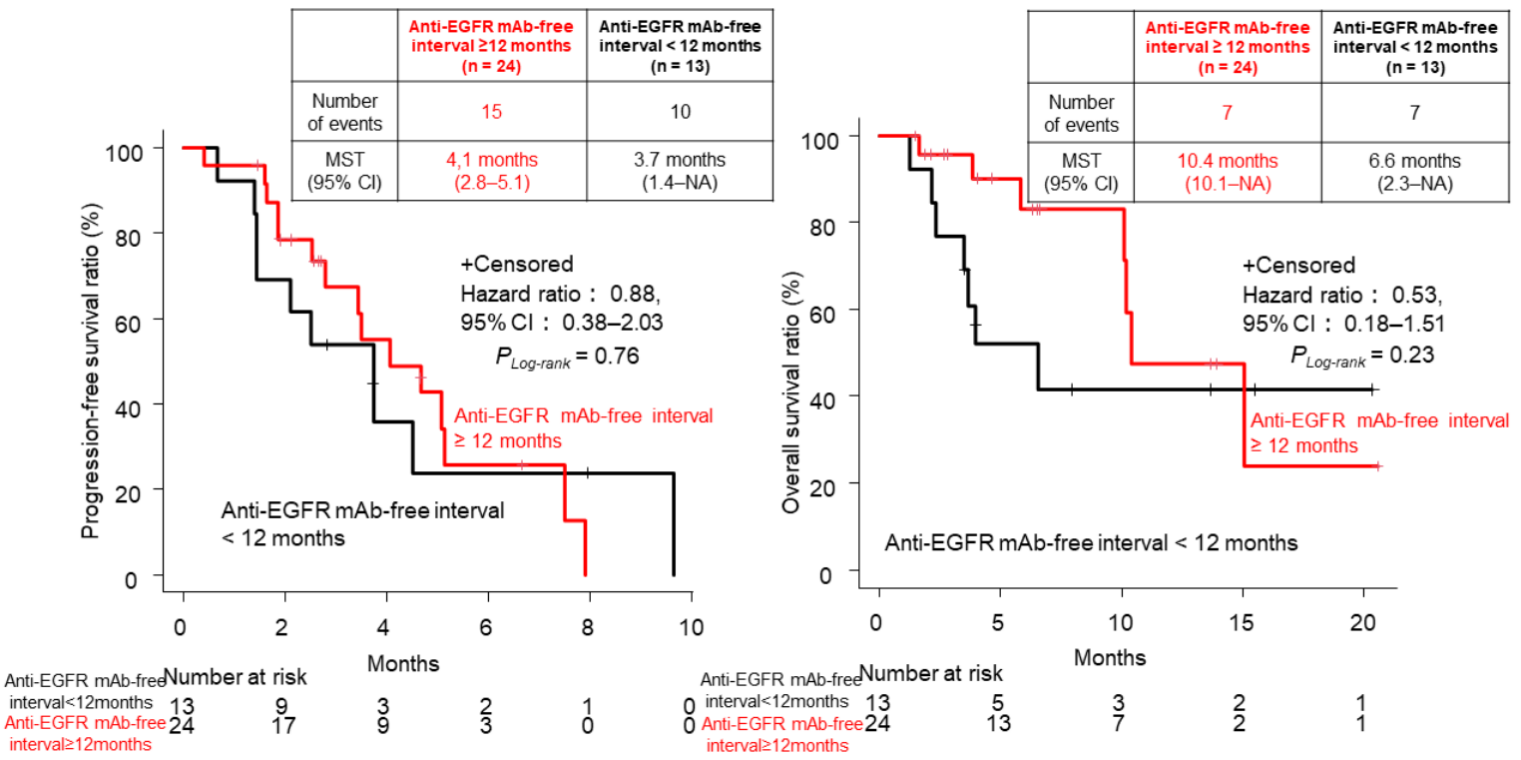
Progression-free survival (PFS) and overall survival (OS) for anti-epidermal growth factor receptor (EGFR) monoclonal antibody (mAb) rechallenge for patients with metastatic colorectal cancer (mCRC) (anti-EGFR mAb-free interval ≥ 12 months or < 12 months) (*n* = 37) MST, median survival time; CI, confidence interval; NA, not applicable

Similarly, patients who had achieved PR during the first-line chemotherapy containing anti-EGFR mAb (*n* = 27) had significantly longer PFS (median: 3.7 vs. 1.5 months; HR, 0.21; 95% CI, 0.08–0.58; *P*_*Log−rank*_ = 0.00087) and longer OS (HR, 0.32; 95% CI, 0.096–1.08; *P*_*Log−rank*_ = 0.052) than those with SD (*n* = 6), albeit with no statistical significance (Fig. 3). However, no significant differences were observed in ORR and DCR between patients achieving PR and those showing SD in first-line chemotherapy (ORR 14.8% vs. 0% *P* = 0.56, DCR 55.6% vs. 16.7% *P* = 0.09) (Supplemental Table 2). Furthermore, no correlation was observed between PFS and aEFI (*r* = -0.0078, *P* = 0.96) nor significant differences in PFS and OS between the two groups divided by the cutoff of 12 months (≥ 12 months [*n* = 24] vs. < 12 months [*n* = 13]; PFS: HR, 0.88; 95% CI, 0.38–2.03, *P*_*Log−rank*_ = 0.76; OS: HR, 0.53; 95% CI, 0.18–1.51, *P*_*Log−rank*_ = 0.23) (Fig. 4, Supplemental Table 4).

### Univariate and multivariate analyses

In the univariate analysis of the 39 patients who received anti-EGFR mAb rechallenge, response during the first-line anti-EGFR mAb treatment was associated with longer PFS (responder vs. non-responder: HR, 0.21; *P* = 0.0025) and relatively longer OS (HR, 0.32; *P* = 0.066), whereas a high CA19-9 level was associated with shorter PFS (HR, 2.36; *P* = 0.041) and shorter OS (HR, 4.04; *P* = 0.034). In the multivariate analysis, response during the first-line chemotherapy containing anti-EGFR mAb was the only significant factor for longer PFS (HR, 0.21; *P* = 0.0026). Patients with high CA19-9 levels had a significantly shorter OS than others (HR, 5.02; *P* = 0.02), and response to first-line anti-EGFR mAb was also an independent prognostic factor of longer OS (HR, 0.23; *P* = 0.026; Table 2).

**Table 2:** Univariate and multivariate analyses of PFS and OS (*n* = 39)

	Univariate analysis				Multivariate analysis			
	HR	Lower 95% CI	Upper 95% CI	*P* value	HR	Lower 95% CI	Upper 95% CI	*P* value
**PFS**								
Age (< 65* or ≥ 65 years)	1.51	0.68	3.31	0.31				
Sex (Female* or Male)	1.05	0.46	2.39	0.91				
Primary resection (not resected* or resected)	0.72	0.29	1.77	0.47				
Liver metastasis (Negative* or Positive)	1.52	0.61	3.82	0.37				
Lung metastasis (Negative* or Positive)	0.99	0.43	2.32	0.99				
Peritoneal metastasis (Negative* or Positive)	0.81	0.35	1.87	0.62				
Lymph node metastases (Negative* or Positive)	1.08	0.47	2.45	0.86				
CEA (ng/mL) (< 5* or ≥ 5)	1.78	0.41	7.69	0.44				
CA19-9 (U/mL) (< 37* or ≥ 37)	2.36	1.04	5.36	0.041	2.35	0.96	5.75	0.062
Number of metastatic organs (< 3* or ≥ 3)	1.53	0.68	3.41	0.3				
Anti-EGFR mAb-free interval (< 12 months or ≥ 12 months)	0.95	0.39	2.33	0.91				
Re-challenge treatment lines (fourth or later* or 3rd)	0.44	0.18	1.11	0.083	0.67	0.2	2.28	0.53
ctDNA *RAS* status (Positive* or Negative)	0.39	0.15	1.02	0.055	0.45	0.17	1.21	0.11
First-line anti-EGFR mAb best response (SD*or PR)	0.21	0.08	0.58	0.0025	0.21	0.078	0.58	0.0026
**OS**								
Age (< 65* or ≥ 65)	1.08	0.37	3.14	0.9				
Sex (Female* or Male)	1.03	0.31	3.40	0.96				
Primary resection (not resected* or resected)	0.55	0.18	1.66	0.29				
Liver metastasis (Negative* or Positive)	1.39	0.38	5.03	0.62				
Lung metastasis (Negative* or Positive)	0.84	0.28	2.52	0.76				
Peritoneal metastasis (Negative* or Positive)	0.5	0.14	1.81	0.29				
Lymph node metastases (Negative* or Positive)	0.91	0.31	2.69	0.87				
CEA (ng/mL) (< 5* or ≥ 5)	1.41	0.18	10.94	0.75				
CA19-9 (U/mL) (< 37* or ≥ 37)	4.04	1.11	14.64	0.034	5.02	1.29	19.5	0.02
Number of metastatic organs (< 3* or ≥ 3)	0.76	0.27	2.2	0.62				
anti-EGFR mAb-free interval (< 12 months* or ≥ 12 months)	0.71	0.24	2.11	0.53				
Re-challenge treatment lines (fourth or later* or 3rd)	0.51	0.14	1.85	0.31				
ctDNA *RAS* status (Positive* or Negative)	0.82	0.23	2.97	0.76				
First-line anti-EGFR mAb best response (SD*or PR)	0.32	0.10	1.08	0.066	0.23	0.064	0.84	0.026

*Reference

Anti-EGFR mAb: Anti-epidermal growth factor receptor monoclonal antibody

CA19-9: Carbohydrate antigen 19 − 9

CEA: Carcinoembryonic antigen

CI: Confidence Interval

ctDNA: Circulating tumor DNA

HR: Hazard ratio

PR: Partial response

SD: Stable disease

*RAS*: Rat sarcoma viral oncogene homolog

PFS: Progression-free survival

OS: Overall survival

## Discussion

In this study, the frequency of ctDNA *RAS* MT was 40.5%, which was associated with high tumor burden such as liver metastasis and high tumor marker levels. On the contrary, Neo*RAS*, which is defined as conversion from initially diagnosed *RAS* MT to WT following treatment, was reportedly associated with the absence of liver metastasis, small tumor diameter, and low tumor markers (Osumi et al. 2023). A previous study reported that ctDNA *RAS* MT tended to be found in patients with liver metastases and significantly larger tumor sizes, and the false negative rate of ctDNA was low (Lim et al. 2021). The study revealed that anti-EGFR mAb rechallenge was less effective in patients with liver metastasis compared with those without (Ciardiello et al. 2024). These patients also have synchronous metastases and significantly higher tumor markers (Lim et al. 2021). Similarly, in our analysis, the rechallenge treatment was ineffective for patients with baseline high CA19-9. Our univariate and multivariate analyses suggested that patients with ctDNA *RAS* MT tended to have a short PFS. These results suggest that ctDNA *RAS* MT detectability may depend on tumor volumes, and ctDNA *RAS* status may be associated with the efficacy of anti-EGFR mAb rechallenge.

Minor *RAS* MT, other than *KRAS* exon2, was detected more frequently than major *RAS* MT. These results are consistent with previous findings (Morelli et al. 2015). Although the mechanism for this finding remains to be determined, the proportion of cancer cells with minor *RAS* MT was extremely small to be detected at the initial diagnosis. *RAS* codon 61 and 146 MT, which have weaker *RAS*-GTPase activity in the transforming assay, might have a lower growth advantage compared with *RAS* exon2 MT. Thereafter, these cancer cells with minor *RAS* MT increase under the stress of anti-EGFR mAb. These minor *RAS* MT may be associated with acquired resistance (Morelli et al. 2015).

In this study, the efficacy of first-line anti-EGFR mAb significantly influenced the clinical outcomes of anti-EGFR mAb rechallenge in clinical practice. Previous studies that enrolled patients who responded to prior chemotherapy containing anti-EGFR mAb as the inclusion criteria showed favorable clinical outcomes of anti-EGFR mAb rechallenge (Cremolini et al. 2019; Sartore-Bianchi et al. 2022). Thus, response to prior chemotherapy containing anti-EGFR mAb may be a clinical marker for predicting the response to anti-EGFR mAb rechallenge as a positive selection. However, considering that responders to prior chemotherapy containing anti-EGFR mAb showed disease progression, some of the acquired resistance should disappear before the anti-EGFR mAb rechallenge. It is not clear what mechanism contributed to the resumed sensitivity for this positive selection.

Ad-hoc analysis of the PURSUIT study, another prospective study of anti-EGFR mAb rechallenge, showed a significantly higher ORR in patients with a longer aEFI than in those with a shorter aEFI (≥ 365 vs. < 365 days; 44.4% vs. 7.3%; *P* = 0.0037) (Kagawa et al. 2022). It is speculated that initially, the major clone with *RAS* WT re-expands during the anti-EGFR mAb-free period. Thus, sufficient aEFI between the end of the last anti-EGFR mAb administration and the first day of anti-EGFR mAb rechallenge is considered a requirement for resuming sensitivity. However, our study showed neither a correlation between PFS and aEFI (*r* = -0.0078, *P* = 0.96) nor significant differences in PFS between the two groups based on a cut-off of 12 months (≥ 12 months [*n* = 24] vs. < 12 months [*n* = 13]; *P*_*Log−rank*_ = 0.76; HR, 0.88; 95% CI, 0.38–2.03). Similarly, in the ad-hoc analysis of the E-rechallenge trial, no significant differences were observed in the ORR, PFS, or OS according to aEFI (Osawa et al. 2018). Furthermore, the CHRONOS study, the first trial to prospectively evaluate the efficacy of anti-EGFR mAb rechallenge based on ctDNA mutational status, showed no correlation between the aEFI and probability of clinical response. As the results for the relationship between rechallenge with anti-EGFR mAb and the aEFI are inconsistent, further studies are warranted.

Conversely, as negative selection, the exclusion of patients with predictive factors for poor response to anti-EGFR mAb, such as *RAS*/*BRAF* MT, may increase the efficacy of chemotherapy containing anti-EGFR mAb (Cremolini et al. 2017; Manca et al. 2021). Considering that responses to anti-EGFR mAb rechallenge were not observed in the non-responders to the prior chemotherapy containing anti-EGFR mAb and the lack of a clear relationship between aEFI and the efficacy of anti-EGFR mAb rechallenge in this study, some mechanisms of primary resistance to the prior anti-EGFR mAb therapy may have persisted in the non-responders before anti-EGFR mAb rechallenge. Regarding the possible mechanisms of primary resistance to anti-EGFR mAb, constitutive activation of tyrosine kinase receptors other than EGFR through uncommon genomic alterations, such as *MAPK* pathway mutations, *HER2* mutations and amplification, *MET* amplification, and rearrangements of *NTRK*, *ROS*, *ALK*, and *RET*, negatively affects susceptibility to EGFR inhibition (Cremolini et al. 2017, Shitara et al. 2024). Therefore, detecting these resistant mechanisms for negative selection for the anti-EGFR mAb rechallenge is important.

In this study, the proportion of patients with ctDNA *RAS* WT who achieved PR was 15.6%, compared to 0% in those with ctDNA *RAS* MT, and patients with ctDNA *RAS* WT had a significantly better prognosis than those with ctDNA *RAS* MT. According to the results of the CRICKET trial, patients with ctDNA *RAS* WT had a significantly longer PFS (median PFS 4.0 vs. 1.9 months; HR, 0.44; 95% CI, 0.18–0.98; *P* = 0.03) and tended to have longer OS than those with ctDNA *RAS* MT (Cremolini et al. 2019). In Japanese clinical trials of anti-EGFR mAb rechallenge, a post-hoc biomarker analysis (JACCRO CC-08/09AR) showed that patients with ctDNA *RAS* MT had significantly shorter PFS and OS than those with ctDNA *RAS* WT prior to the anti-EGFR mAb rechallenge (mPFS: 2.3 vs. 4.7 months; HR, 6.2; *P* = 0.013; mOS: 3.8 vs. 16.0 months; HR, 12.4; *P* = 0.0028) (Sunakawa et al. 2020). Similarly, in E-rechallenge, another Japanese clinical trial on anti-EGFR mAb rechallenge, the post-hoc analysis showed that the RR of patients with ctDNA all WT (50%) was higher than that of patients with any MT (*KRAS* G12/G13/A59/Q61, *BRAF* V600E, and *EGFR* S492R) (Osawa et al. 2018) (Supplemental Table 5). These results suggest that anti-EGFR mAb rechallenge is less effective in patients with any gene alterations related to the EGFR pathway, including *RAS* detected in ctDNA prior to anti-EGFR mAb, as these gene alterations may be associated with acquired resistance to anti-EGFR mAb. ctDNA test can be used to monitor real-time mutational status compared with tissue biopsy, facilitating the selection of appropriate chemotherapy, including anti-EGFR mAb rechallenge. In addition, this ctDNA test might be beneficial for patients with lesions in areas that are difficult to biopsy.

Regarding treatment lines of anti-EGFR mAb rechallenge in clinical practice, several studies have been reported. The VELO trial is a randomized phase II trial that compared anti-EGFR mAb rechallenge with a standard of care (trifluridine/tipiracil: FTD/TPI), which showed a significant improvement in PFS (Napolitano S et al. 2023). The study did not reveal significant efficacy in OS because of crossover, but a subgroup analysis was performed for patients treated with standard of care who received active anticancer treatment in the fourth-line after progression from FTD/TPI, and the median OS from the start of fourth line therapy was significantly longer in patients treated with anti-EGFR mAb rechallenge than the OS in patients with other therapies (Napolitano S et al. 2023). Therefore, anti-EGFR mAb rechallenge is considered a promising later-line treatment for patients with *RAS*/*BRAF* WT mCRC.

Compared with FTD/TPI plus bevacizumab, which is considered the standard third-line treatment for patients with mCRC (Prager et al. 2023), the ORR of anti-EGFR mAb rechallenge tends to be high, especially for patients with mCRC with ctDNA *RAS* WT. The ongoing PULSE trial is a randomized, phase II, open-label trial comparing the OS of panitumumab rechallenge with standard of care (FTD/TPI or regorafenib) in patients with mCRC without any alterations confirmed with liquid biopsies. The CITRIC trial is a multicenter, randomized, open-labeled, parallel-group, phase II study that evaluated the efficacy and safety of cetuximab plus irinotecan rechallenge versus investigators’ choice in the third-line setting. In addition, the FIRE-4 study is a randomized phase III study that tests the efficacy of early switch maintenance during first line therapy and also investigates rechallenge with cetuximab in later-line treatment using biopsy tissues (Holch et al. 2016). On the contrary, the PARERE study is a randomized phase II study of panitumumab rechallenge, followed by regorafenib versus the reverse sequence in patients with *RAS*/*BRAF* WT refractory mCRC (Moretto et al. 2021), and the CAVE-2 trial is a non-profit phase II, randomized study of the combination of avelumab plus cetuximab as a rechallenge strategy, compared with cetuximab alone in pre-treated patients with *RAS*/*BRAF* WT mCRC (Napolitano et al. 2022); the *RAS* status was examined with ctDNA in the two studies. These ongoing head-to-head clinical trials will optimize treatment strategies in the salvage-line setting for patients with tissue *RAS/BRAF* WT mCRC.

This study has several limitations. First, it was a retrospective study with a small sample size, which might affect the generalizability of the findings; the retrospective nature may also introduce potential selection and information biases. Besides, the potential risk of bias needs to be considered because of the very small number of patients in our subgroup analysis. Some results of our analysis had a *P*-value close to the statistical significance threshold, and they need to be interpreted cautiously. The relatively high frequency of false-negative rates in the BEAMing analysis for patients with mCRC needs to be considered (Bando et al. 2019). Previous studies have highlighted the cutoff for patients with peritoneal metastases alone with a lesion diameter < 20 mm, lung metastases alone with a lesion diameter < 20 mm, or < 10 lesions in total (Vidal et al. 2017; Bando et al. 2019; Kagawa et al. 2021). Therefore, an accurate interpretation of the results of this BEAMing analysis is crucial.

In conclusion, the incidence of ctDNA *RAS* MT mCRC prior to salvage-line treatment was 40.5%, and liver metastases and high tumor volumes (non-resected primary tumor and high tumor markers) were associated with the appearance of ctDNA *RAS* MT mCRC. Rechallenge with anti-EGFR mAb may be effective for patients without *RAS* MT detected in ctDNA and those who respond to first-line anti-EGFR mAb, while no patients with *RAS* MT in ctDNA responded to anti-EGFR mAb rechallenge. This anti-EGFR mAb rechallenge strategy may be a feasible option for patients with mCRC as a late-line treatment, and we need to validate this result prospectively in the future.

## Electronic supplementary material

Below is the link to the electronic supplementary material.

Supplementary Material 1

## Data Availability

The datasets generated and/or analyzed during the current study are not publicly available but are available from the corresponding author upon reasonable request.

## Abbreviations

aEFI: Anti-EGFR mAb-free interval
BRAF: V-raf murine sarcoma viral oncogene homolog B1
CA19-9: Carbohydrate antigen 19 − 9
CEA: Carcinoembryonic antigen
CI: confidence interval
CR: Complete response
ctDNA: Circulating tumor DNA
DCR: Disease control rate
EGFR: Epidermal growth factor receptor
FTD/TPI: trifluridine/tipiracil
HR: Hazard ratio
mAb: Monoclonal antibody
mCRC: Metastatic colorectal cancer
MT: Mutant type
ORR: Objective response rate
OS: Overall survival
mOS: Median overall survival
PD: Progressive disease
PFS: Progression-free survival
mPFS: Median progression-free survival
PR: Partial response
PR: Partial response
RAS: Rat sarcoma viral oncogene homolog
RR: Response rate
SD: Stable disease
SD: Stable disease
WT: Wild-type
