# Supplementary material for: Efficacy of anti-epidermal growth factor antibody rechallenge in RAS/BRAF wild-type metastatic colorectal cancer: a multi-institutional observational study
Source: J Cancer Res Clin Oncol. 2024 Jul 27;150(7):369. doi: 10.1007/s00432-024-05893-1 (PMC11283376; doi:10.1007/s00432-024-05893-1)
Supplement: Supplementary file 1 — Supplementary Material 1 [file 432_2024_5893_MOESM1_ESM.docx]

**Supplemental Table 1. Patient characteristics and response rate/disease control rate of patients treated with anti-EGFR mAb rechallenge (n = 39)**

| **Characteristics, n (%)** | **Total number of patients (n = 39)** |
| --- | --- |
| **Age at enrollment, years** |  |
| Median [range] | 63 [36–82] |
| **Sex** |  |
| Male | 25 (64.1) |
| Female | 14 (35.9) |
| **Primary site** |  |
| Right-sided colon | 3 (7.7) |
| Left-sided colon | 36 (92.3) |
| **Metastatic site** |  |
| Liver |  |
| Present | 28 (71.8) |
| Absent | 11 (28.2) |
| Lung |  |
| Present | 27 (69.2) |
| Absent | 12 (30.8) |
| Lymph node |  |
| Present | 17 (43.6) |
| Absent | 22 (56.4) |
| Peritoneum |  |
| Present | 14 (35.9) |
| Absent | 25 (64.1) |
| **Resection of primary tumor** |  |
| Yes | 27 (69.2) |
| No | 12 (30.8) |
| **Rechallenge treatment lines** |  |
| Third | 14 (35.9) |
| Fourth | 13 (33.3) |
| Fifth | 7 (17.9) |
| Sixth | 4 (10.3) |
| Ninth | 1 (2.6) |
| **Anti-EGFR mAb free interval, Median [range], (months)** | 14.9 [5.1–82.6] |
| **Response of first-line anti-EGFR mAb** |  |
| Partial response | 27 (69.2) |
| Stable disease | 6 (15.4) |
| Progressive disease | 0 (0) |
| Not evaluable | 6 (15.4) |
| **Tumor markers** |  |
| CEA median, [range] (ng/mL) | 90.8 [2.2–3720.2] |
| CA19-9 median, [range] (U/mL) | 55 [1.0–6927.8] |
| **Median PFS, months (95% CI)** | 3.7 (2.5–5.1) |
| **Median OS, months (95% CI)** | 10.4 (6.6–NA) |

anti-EGFR mAb: Anti-epidermal growth factor receptor monoclonal antibody

CA19-9: Carbohydrate antigen 19-9

CEA: Carcinoembryonic antigen

OS: Overall survival

PFS: Progression-free survival

NA: Not applicable

**Supplemental Table 2. Patient characteristics and response rate/disease control rate of patients treated with anti-EGFR mAb rechallenge (PR or SD as best response in first-line anti-EGFR mAb) (n = 33)**

| **Characteristics, n (%)** | **PR in first line (n = 27, 81.8%)** | **SD in first line (n = 6, 18.2%)** | ***P* value** |
| --- | --- | --- | --- |
| **Age at enrollment, years** |  |  |  |
| Median [range] | 62.5 [42–82] | 62 [54–73] | 0.54 |
| **Sex** |  |  |  |
| Male | 19 (70.4) | 4 (66.7) | 1 |
| Female | 8 (29.6) | 2 (33.3) |  |
| **Primary site** |  |  |  |
| Right-sided colon | 3 (11.1) | 0 (0) | 1 |
| Left-sided colon | 24 (88.9) | 6 (100) |  |
| **Metastatic site** |  |  |  |
| Liver |  |  |  |
| Present | 22 (81.5) | 4 (66.7) | 0.16 |
| Absent | 5 (18.5) | 2 (33.3) |  |
| Lung |  |  |  |
| Present | 22 (81.5) | 3 (50.0) | 0.14 |
| Absent | 5 (18.5) | 3 (50.0) |  |
| Lymph node |  |  |  |
| Present | 13 (48.1) | 2 (33.3) | 1 |
| Absent | 14 (51.9) | 4 (66.7) |  |
| Peritoneum |  |  |  |
| Present | 12 (44.4) | 3 (50.0) | 0.64 |
| Absent | 15 (55.6) | 3 (50.0) |  |
| **Resection of primary tumor** |  |  |  |
| Yes | 18 (66.7) | 5 (83.3) | 0.64 |
| No | 9 (33.3) | 1 (16.7) |  |
| **Rechallenge treatment lines** |  |  |  |
| Third | 11 (40.8) | 0 (0) | 0.05 |
| Fourth | 8 (29.6) | 3 (50.0) |  |
| Fifth | 4 (14.8) | 2 (33.3) |  |
| Sixth | 4 (14.8) | 0 (0) |  |
| Ninth | 0 (0) | 1 (16.7) |  |
| **Tumor markers** |  |  |  |
| CEA, median [range] (ng/mL) | 90.55 [3.0–3720.7] | 94.6 [2.5–118.4] | 0.36 |
| CA19-9, median [range] (U/mL) | 36.65 [1.0–5184.5] | 67.7 [3.9–6927.8] | 0.1 |
| **Best overall response** |  |  |  |
| Complete response | 0 (0) | 0 (0) |  |
| Partial response | 4 (14.8) | 0 (0) |  |
| Stable disease | 11 (40.8) | 1 (16.7) |  |
| Progressive disease | 8 (29.6) | 5 (83.3) |  |
| Not evaluated | 4 (14.8) | 0 (0) |  |
| **Response rate, n (%)** | 4 (14.8) | 0 (0) | 0.56 |
| **Disease control rate, n (%)** | 15 (55.6) | 1 (16.7) | 0.09 |

Anti-EGFR mAb: Anti-epidermal growth factor receptor monoclonal antibody

CA19-9: Carbohydrate antigen 19-9

CEA: Carcinoembryonic antigen

PR: Partial response

SD: Stable disease

**Supplemental Table 3. Patient characteristics and response rate/disease control rate of anti-EGFR mAb rechallenge for mCRC patients with ctDNA *RAS* WT or MT (n = 39)**

| **Characteristics, n (%)** | **ctDNA *RAS* WT (n = 32, 82.1%)** | **ctDNA *RAS* MT (n = 7, 17.9%)** | ***P* value** |
| --- | --- | --- | --- |
| **Age at enrollment, years** |  |  |  |
| Median [range] | 62 [36–82] | 66 [59–78] | 0.27 |
| **Sex** |  |  |  |
| Male | 18 (61.4) | 7 (100) | 0.036 |
| Female | 14 (38.6) | 0 (0) |  |
| **Primary site** |  |  |  |
| Right-sided colon | 2 (6.3) | 1 (14.3) | 0.46 |
| Left-sided colon | 30 (93.7) | 6 (85.7) |  |
| **Metastatic site** |  |  |  |
| Liver |  |  |  |
| Present | 21 (65.6) | 7 (100) | 0.16 |
| Absent | 11 (34.4) | 0 (0) |  |
| Lung |  |  |  |
| Present | 22 (68.8) | 5 (71.4) | 1 |
| Absent | 10 (31.2) | 2 (28.6) |  |
| Lymph node |  |  |  |
| Present | 13 (40.6) | 4 (57.1) | 0.68 |
| Absent | 19 (59.4) | 3 (42.9) |  |
| Peritoneum |  |  |  |
| Present | 12 (37.5) | 2 (28.6) | 1 |
| Absent | 20 (62.5) | 5 (71.4) |  |
| **Resection of primary tumor** |  |  |  |
| Yes | 25 (78.1) | 2 (28.6) | 0.02 |
| No | 7 (21.9) | 5 (71.4) |  |
| **Rechallenge treatment lines** |  |  |  |
| Third | 12 (37.5) | 2 (28.6) | 1 |
| Fourth | 10 (31.2) | 3 (42.8) |  |
| Fifth | 7 (21.9) | 0 (0) |  |
| Sixth | 3 (9.4) | 1 (14.3) |  |
| Ninth | 0 (0) | 1 (14.3) |  |
| **Tumor markers** |  |  |  |
| CEA, median [range] (ng/mL) | 64.25 [2.2–3023.6] | 128.6 [2.5–3720.7] | 0.028 |
| CA19-9, median [range] (U/mL) | 32.45 [1.0–6927.8] | 288.0 [2.0–818] | 0.67 |
| **Best overall response** |  |  |  |
| Complete response | 0 (0) | 0 (0) |  |
| Partial response | 5 (15.7) | 0 (0) |  |
| Stable disease | 13 (40.6) | 2 (28.6) |  |
| Progressive disease | 10 (31.2) | 4 (57.1) |  |
| Not evaluated | 4 (12.5) | 1 (14.3) |  |
| **Response rate, (%)** | 5 (15.6) | 0 (0) | 0.56 |
| **Disease control rate, (%)** | 18 (56.2) | 2 (28.6) | 0.22 |

Anti-EGFR mAb: Anti-epidermal growth factor receptor monoclonal antibody

CA19-9: Carbohydrate antigen 19-9

CEA: Carcinoembryonic antigen

ctDNA: Circulating tumor DNA

WT: Wild type

MT: Mutant type

*RAS*: Rat sarcoma viral oncogene homolog

mCRC: metastatic colorectal cancer

**Supplemental Table 4. Patient characteristics and response rate/disease control rate of anti-EGFR mAb rechallenge for mCRC patients with anti-EGFR mAb-free interval ≥12 months or <12 months (n = 37)**

| **Characteristics, n (%)** | **Anti-EGFR mAb-free  interval ≥ 12 months  (n = 24, 64.9%)** | **Anti-EGFR mAb-free interval < 12 months  (n = 13, 35.1%)** | ***P* value** |
| --- | --- | --- | --- |
| **Age at enrollment, years** |  |  |  |
| Median [range] | 65 [36–82] | 63 [43–78] | 0.55 |
| **Sex** |  |  |  |
| Male | 14 (58.3) | 10 (76.9) | 0.31 |
| Female | 10 (41.7) | 3 (23.1) |  |
| **Primary site** |  |  |  |
| Right-sided colon | 1 (4.2) | 2 (15.4) | 0.28 |
| Left-sided colon | 23 (95.8) | 11 (84.6) |  |
| **Metastatic site** |  |  |  |
| Liver |  |  |  |
| Present | 15 (62.5) | 11 (84.6) | 0.26 |
| Absent | 9 (37,5) | 2 (15.4) |  |
| Lung |  |  |  |
| Present | 17 (70.8) | 9 (69.2) | 1 |
| Absent | 7 (29.2) | 4 (30.8) |  |
| Lymph node |  |  |  |
| Present | 8 (33.3) | 7 (53.8) | 0.3 |
| Absent | 16 (66.7) | 6 (46.2) |  |
| Peritoneum |  |  |  |
| Present | 9 (37.5) | 4 (30.8) | 0.73 |
| Absent | 15 (62.5) | 9 (69.2) |  |
| **Resection of primary tumor** |  |  |  |
| Yes | 17 (70.8) | 9 (69.2) | 1 |
| No | 7 (29.2) | 4 (30.8) |  |
| **Rechallenge treatment lines** |  |  |  |
| Third | 10 (41.7) | 3 (23.1) | 0.31 |
| Fourth | 6 (25.0) | 6 (46.2) |  |
| Fifth | 5 (20.8) | 2 (15.3) |  |
| Sixth | 3 (12.5) | 1 (7.7) |  |
| Ninth | 0 (0) | 1 (7.7) |  |
| **Tumor markers** |  |  |  |
| CEA median, [range] (ng/mL) | 90.8 [2.2–839.9] | 90.8 [3.0–3720.7] | 0.14 |
| CA19-9 median, [range] (U/mL) | 42.3 [1.0–1379] | 288 [2.0–6927.8] | 0.034 |
| **Best overall response** |  |  |  |
| Complete response | 0 (0) | 0 (0) |  |
| Partial response | 4 (16.7) | 1 (7.6) |  |
| Stable disease | 9 (37.5) | 6 (46.2) |  |
| Progressive disease | 7 (29.1) | 6 (46.2) |  |
| Not evaluated | 4 (16.7) | 0 (0) |  |
| **Response rate, (%)** | 4 (16.7) | 1 (7.6) | 0.64 |
| **Disease control rate, (%)** | 13 (54.2) | 7 (53.8) | 1 |

Anti-EGFR mAb: Anti-epidermal growth factor receptor monoclonal antibody

CA19-9: cancer antigen 19-9

CEA: Carcinoembryonic antigen

mCRC: metastatic colorectal cancer

**Supplemental Table 5. Previous reports of anti-EGFR mAb rechallenge**

| **Study name** | **Phase** | **Assay** | **Number** | **Primary endpoint** | **Regimen** | **ORR (%)　(All: ctDNA *RAS* WT)** | | **mPFS (months) (All: ctDNA *RAS* WT)** | | **mOS (months) (All: ctDNA *RAS* WT)** | |
| --- | --- | --- | --- | --- | --- | --- | --- | --- | --- | --- | --- |
| CRICKET | Single-arm phase II | Droplet digital PCR | 28 (ctDNA: 25) | ORR | Cmab+CPT-11 | 21.4 | 31 (n = 13) | 3.4 | 4 | 9.8 | 12.5 |
| E-rechallenge | Single-arm phase II | Droplet digital PCR | 33 (ctDNA: 24) | ORR | Cmab+CPT-11 | 15.6 | 50 (n = 24) | 2.97 | 7 | 8.6 | N/A |
| JACCRO CC-08 | Single-arm phase II | BEAMing (OncoBEAM^TM^ *RAS* CRC KIT) | 34 | 3-month PFS | Cmab+CPT-11 | 2.9 | N/A | 2.4 | N/A | 8.1 | N/A |
| JACCRO CC-09 | Single-arm phase II | BEAMing (OncoBEAM^TM^ *RAS* CRC KIT) | 25 | 3-month PFS | Pmab+CPT-11 | 8.3 | N/A | 3.1 | N/A | 8.9 | N/A |
| CAVE | Single-arm phase II | Quantitative PCR | 77 (ctDNA: 67) | OS | Avelumab+Cmab | 7.8 | 8.5 | 3.6 | 4.1 | 11.6 | 15.7 |
| CHRONOS | Single-arm phase II | Droplet digital PCR | 27 | ORR | Pmab | 30 | 30 | 16.4 | 16.4 | NR | NR |
| PURSUIT | Single-arm phase II | BEAMing (OncoBEAM^TM^ *RAS* CRC KIT) | 50 | ORR | Pmab+CPT-11 | 14 | 14 | 3.6 | 3.6 | NR | NR |
| VELO | Randomized phase II | Real-time PCR  (Idylla^TM^) | 62 | PFS | Pmab+FTD/TPI | 9.7 | 13.3 | 4 | 6.4 | 13.1 | 14 |

Anti-EGFR mAb: Anti-epidermal growth factor receptor monoclonal antibody

PCR: Polymerase chain reaction

ctDNA: Circulating tumor DNA

CPT-11: Irinotecan

BEAMing: Beads, emulsion, amplification, magnetics

*RAS*: Rat sarcoma viral oncogene homolog

WT: wild type

Cmab: Cetuximab

Pmab: Panitumumab

N/A: Not applicable

FTD/TPI: Trifluridine/tipiracil

ORR: Objective response rate

mOS: median overall survival

mPFS: median progression-free survival

NR: Not recorded
